# Relationship between pancreatic cancer resection rate and survival at population level: systematic review

**DOI:** 10.1093/bjsopen/zraf007

**Published:** 2025-03-25

**Authors:** Elizabeth B Lockie, Amy Sylivris, Sanjay Pandanaboyana, John Zalcberg, Anita Skandarajah, Benjamin P Loveday

**Affiliations:** Department of Surgery, The University of Melbourne, Parkville, Victoria, Australia; Department of General Surgical Specialties, The Royal Melbourne Hospital, Parkville, Victoria, Australia; Department of General Surgical Specialties, The Royal Melbourne Hospital, Parkville, Victoria, Australia; Hepato-Pancreatico-Biliary Centre, Freeman Hospital, Newcastle upon Tyne, UK; Population Health Sciences Institute, Newcastle University, Newcastle upon Tyne, UK; Department of Surgery, The University of Melbourne, Parkville, Victoria, Australia; School of Public Health, Faculty of Medicine, Monash University, Clayton, Victoria, Australia; Department of Medical Oncology, Alfred Health, South Yarra, Victoria, Australia; Department of Surgery, The University of Melbourne, Parkville, Victoria, Australia; Department of General Surgical Specialties, The Royal Melbourne Hospital, Parkville, Victoria, Australia; Department of Surgery, Peter MacCallum Cancer Centre, Parkville, Victoria, Australia; Department of Surgery, The University of Melbourne, Parkville, Victoria, Australia; Department of General Surgical Specialties, The Royal Melbourne Hospital, Parkville, Victoria, Australia; Department of Surgery, Peter MacCallum Cancer Centre, Parkville, Victoria, Australia

## Abstract

**Background:**

Surgery combined with chemotherapy provides the best chance of survival in pancreatic cancer. This study investigated whether increasing the resection rate at a population level improves overall survival and modelled the interaction between resection rate, perioperative mortality rate, and population survival.

**Methods:**

A systematic review was conducted on studies reporting resection rate and survival outcomes in patients with pancreatic cancer at a population level. MEDLINE, Embase and Evidence-Based Medicine Reviews were searched up to February 2024. The primary outcome was overall population-level survival. A model for 1-year survival incorporating varying resection and perioperative mortality rates was developed.

**Results:**

The search identified 3967 studies; 19 were eligible (516 789 patients). A significant association was observed between resection rate and pancreatic cancer population survival at 1 year (r^2^ = 0.46, *P* = 0.001). A weak but significant association was noted between resection rate and (neo)adjuvant chemotherapy (r^2^ = 0.26, *P* = 0.03). One-year pancreatic cancer population survival was significantly associated with chemotherapy (r^2^ = 0.63; *P* = 0.004), but the effect was weaker than for resection rate (regression slope 0.26 *versus* 0.94 respectively). According to the developed model, for example, increasing the resection rate from 10 to 15% and perioperative mortality rate from 2 to 3% would lead to a 1-year survival increase from 17.6% to 22.1%.

**Conclusion:**

A higher resection rate at a population level was associated with improved survival of the pancreatic cancer population. While some of this benefit was linked to increasing (neo)adjuvant chemotherapy use, the effect of resection rate was stronger. Strategies to enhance the resection rate at national and regional levels should be explored. Establishing a benchmark for resection rate could support patient-centred healthcare and promote equitable access to high-quality pancreatic cancer care.

## Teaser

The resection rate for pancreatic cancer may improve survival of the overall pancreatic cancer population, potentially through optimizing adjunctive care. Defining a benchmark for resection rate aligns with patient-centred healthcare and may ensure equitable access to high-quality pancreatic cancer care.

## Introduction

Pancreatic cancer has the highest mortality rate among common cancer types, with a 5-year survival rate of 5–13%^1,2^. Globally, pancreatic cancer is the 12th most common cancer; however, it ranks 4th for cancer-related deaths in Australia and the USA^2,3^. Since 1990, the global age-standardized incidence of pancreatic cancer has doubled. However, the current decrease in the death-to-incidence ratio suggests an overall improvement in care^4,5^. Surgery with chemotherapy is associated with the longest survival^6^, although it is mostly limited to patients with localized disease (stage 1–2) and good performance status^7^. Advances in neoadjuvant therapies and a more radical surgical approach has enabled patients with borderline resectable or locally advanced (stage 3) disease to potentially benefit from resection^8–11^, and even highly selected patients with metastatic (stage 4) disease^12^. However, pancreatic surgery has a morbidity rate of 22–40% and a perioperative mortality rate of 2–9%^13,14^, therefore considered surgical planning is important.

Resection rates vary between regions, ranging from 35 to 69% in resectable cancers^15^. Given the significant survival benefit associated with surgery, low resection rates may disadvantage patients at a population level^6^. In one study in the USA, only 38.2% of patients with resectable stage 1 disease had surgery despite an absence of an identifiable surgical contraindication, such as patient co-morbidities or refusal of surgery, and this low resection rate likely decreased the median survival for the entire cohort of patients with stage 1 disease^16^. The reasons for varied resection rates in this study and others are multiple. At a centre level, higher resection rates tend to occur in higher volume centres^16,17^ as they may resect more advanced cancers and higher risk patients due to increased experience in extended resections and capacity to rescue patients who sustain a complication^13^. At these specialized centres, rates of neoadjuvant therapy may also be higher^16,18^, thus expanding the pool of potential surgical candidates. At a regional or national level, variations in resection rates are driven by factors such as differing stages of disease at diagnosis, geographical access to pancreatic surgery centres^19^, socioeconomic barriers, racial disparities, healthcare delivery models and patient acceptance of resection^16,20,21^.

Studies have shown that a higher resection rate at a regional or system level are associated with longer survival for the overall pancreatic cancer population^22,23^. However, inappropriately liberal patient selection and/or lower rescue rates could increase perioperative mortality rates, which would adversely affect long-term survival, in addition to being medically futile^24^. The aim of this systematic review was to characterize the relationship between pancreatic cancer resection rates, chemotherapy use and survival for patients with pancreatic cancer (including resected and non-resected patients) at a population level. A further objective was to develop a model to estimate the effect of a range of resection and perioperative mortality rates on pancreatic cancer survival for the entire disease population.

## Methods

A systematic review with a meta-analysis was conducted. This study followed the Preferred Reporting Items for Systematic Reviews and Meta-Analyses (PRISMA) guidelines^25^.

### Eligibility criteria

The review included studies based on population-level data (national, state, province or other regional administrative jurisdictions) that reported a resection rate and survival data within an overall cohort of patients with pancreatic cancer (all disease stages, resected and non-resected patients). Studies including patients with other diagnoses (for example ampullary, bile duct or gallbladder cancer) were excluded unless data for patients with pancreatic cancer could be separated. The histopathology of interest was pancreatic ductal adenocarcinoma (PDAC). Studies that did not specify the type of pancreatic cancer were included in the review, with notation for this. Studies based on institutional or hospital network data were excluded, as they do not represent population-level data. Chemotherapy rates were considered a cofactor for survival along with surgery at a population level, and therefore chemotherapy rates were extracted where available. If not reported along with resection rates, then publications or administrative data for the same regions and time intervals were identified to extract chemotherapy rates. Studies that were restricted to patients in specific age brackets (50 years of age or younger or 80 years of age or older) were excluded, as well as studies in languages other than English, reviews, case reports and publications with only abstracts if the abstract did not provide sufficient information. There was no limit on time interval of the publication or data.

### Search strategy

MEDLINE (up to February 2024), Embase (up to February 2024) and Evidence-Based Medicine Reviews (up to January 2024) were searched with the assistance of a health sciences librarian. The search strategy included terms for pancreatic cancer, pancreatoduodenectomy, surgery and survival rate (full search terms in *Supplementary methods*). References of identified articles were screened for other relevant articles.

### Study selection

The literature search results were uploaded to the Covidence systematic review web-based tool^26^. Duplicates were removed using Covidence and manually by the reviewers. Two investigators (E.L., A.S.) independently screened title and abstracts against inclusion criteria, then screened full articles to ensure eligibility. Discrepancies around article inclusion were reviewed by a third investigator (B.L.) and inclusion/exclusion based on consensus.

### Data items and extraction

Data about resection rate and survival of the entire pancreatic cancer population (median overall survival (OS) in months, or survival rate at 1, 3 or 5 years) were extracted. When reported, data regarding percentage of patients with metastatic disease at diagnosis, perioperative mortality rates (in-hospital, 30- or 90-day mortality rates), and chemotherapy rates in the resected and non-resected patients was extracted. Resection was defined as any surgical procedure aimed at removing pancreatic cancer, irrespective of final margin status. Chemotherapy was categorized as (neo)adjuvant for resected patients and palliative for non-resected patients. Total chemotherapy included both (neo)adjuvant and palliative chemotherapy.

### Risk of bias

The risk of bias was assessed with the Risk Of Bias In Non-randomised Studies of Interventions (ROBINS-I) tool^27^ for non-randomized studies, with the outcome of interest being survival. The risk of bias within each domain was considered to be due to: bias due to confounding (from disease stage and chemotherapy), bias due to selection of participants (from study including histopathology other than PDAC), bias in classification of intervention (due to study including resection as all pancreatectomy types) and bias due to missing data. Domains such as bias due to deviations from intended interventions, outcome measurement and selective reporting were not assessed as they were not relevant in this review. One investigator (E.L.) assessed the risk of bias, grading each domain as low, moderate, serious, critical or no information, with an overall grade for each study. In the ROBINS-I tool, low risk of bias means the study is comparable to a randomized trial, with moderate risk meaning the study is sound for a non-randomized trial. All studies were weighted equally.

### Data analysis

Characteristics and findings of the included studies were summarized in tabular form. The pooled mean resection rate was calculated using a random-effects model. The relationship between resection rates, chemotherapy rates ((neo)adjuvant, palliative, overall) and survival (median OS, 1-year, 3-year, 5-year) was analysed using linear regression with results reported as r^2^. Secondary analyses of resection rate *versus* chemotherapy and perioperative mortality rate were completed. A subset analysis of studies with a date range of less than 5 years was conducted to explore possible trends in treatment regimens and survival over time. A model examining resection rate, perioperative mortality rate and 1-year survival was constructed using linear regression data from the 1-year survival analysis. This was used to model the number of patients who would survive at 1 year from diagnosis, depending on resection rates of 5–30% (5% increments) and perioperative mortality rates from 1–10% (1% increments), based on a starting population of 1000 patients with pancreatic cancer.

## Results

The search strategy study identified 3967 studies after deduplication. After screening titles and abstracts, 66 full-text articles were assessed for eligibility and 47 excluded, leaving 19 studies included in the final analysis. *Figure S1* shows reason for exclusion and the study selection flow chart.

The 19 included studies (*Table 1*)^1,15,22,28–43^ were derived from registries in the USA (*n* = 6), Australia (*n* = 3), Sweden (*n* = 2), Italy (*n* = 2), The Netherlands (*n* = 2), Norway (*n* = 2), Canada (*n* = 2), New Zealand (*n* = 1), Czech Republic (*n* = 1), Denmark (*n* = 1), Belgium (*n* = 1) and Slovenia (*n* = 1). Two studies were multinational studies^15,38^. The registries the studies were based on are shown in *Table 1*, with four studies using data linkage from multiple sources. Eleven studies included only PDAC, three included pancreatic cancer excluding neuroendocrine tumours (NETs), one had 68.4% PDAC (and 26.4% unknown, 2.1% mucinous, 3.1% other) and four did not specify beyond ‘pancreatic cancer’.

**Table 1:** Studies meeting inclusion criteria

						Chemotherapy*			Survival				
Study	Location Study years Registry	Number ofpatients	Inclusion	Metastatic (%)	Resection rate (%)	Non-resected patients (%)	Resected patients (%)	Periop. mortality rate (%)	Median (months)	1-year (%)	3-year (%)	5-year (%)	ROBINS-I
Tingstedt (2019)^34^	Sweden 2010–2016 Swedish National Periampullary and Pancreatic Cancer registry	6891	PDAC	>50	21.6	–	–	30-day: 1.5 90-day: 3.5	–	–	–	6	M
Salami (2019)^35^	USA 2004–2014 SEER	62 201	PDAC	58.8	13.3	–	–	–	5	–	–	–	M
	USA 2004–2009 SEER	30 203	PDAC	59.4	14.1	–	–	–	4	–	–	–	
	USA 2010–1014 SEER	31 998	PDAC	58.2	12	–	–	–	5	–	–	–	
Linder (2007)^36^	Sweden 1980–2000 Crosslinkage of Swedish Hospital Discharge Register, Cancer Register & Register of Causes of Death	16 758	PDAC	–	10.9	–	–	–	3	12	–	1.4	M
Kirkegard (2022)^41^	Denmark 1980–2019 Crosslinkage of Danish National Patient Registry, Civil Registration System, Cancer Registry	20 743	Pancreatic cancer (PDAC 68.4%, unknown 26.4%, mucinous 2.1%, other 3.1%)	45.2	12.8	–	–	–	–	–	–	–	M
	Denmark 1996–2003 As above	5795		36.7	6	–	2.6	–	3.2	–	–	–	
	Denmark 2004–2010 As above	6207		49.2	8.6	32.6	55.6	–	3.4	–	–	–	
	Denmark 2011–2018 As above	8741		47.9	15.9	37.1	72	–	5	–	–	–	
Wakeman (2004)^40^	New Zealand 1994–1997 New Zealand National Cancer Registry	935	Pancreatic cancer	–	7.5	–	–	90-day: 16	3.1	–	–	–	S
Speer (2012)^39^	Australia 2002–2003 Crosslinkage of Victorian Cancer Registry and Registry of Births, Deaths and Marriages	763 616 head & neck 147 body & tail	Pancreatic cancer, excluded NET and ampulla of Vater tumours	–	11.4	–	–	30-day: 5.3	4.5	–	–	2.6	M
Saadat (2024)^38^	Canada 2006–2015 Ontario Cancer Registry	11 512	Pancreatic cancer, excluded NET Age ≥66 years	–	12.5	19	50.8	30-day: 3.3 90-day: 7.1	–	–	7	2.6	S
	USA 2006–2015 SEER	38 858		–	13.4	34.3	66.9	30-day: 4.9 90-day: 11	–	–	4.1	1.3	
Huang (2018)^15^	Multiple countries 2003–2017		PDAC	*Stage III & IV*									M
	USA 2004–2013 SEER	86 466	PDAC	*63.4*	17.7	–	–	30-day: 4	–	25.4	–	4.7	
	Netherlands 2003–2014 Netherlands Cancer Registry	19 684	PDAC	*71*	14.8	18.8	40 Neoadjuvant CTx: 2.2	30-day: 3.1	–	21	–	3.5	
	Belgium 2004–2013 Belgian Cancer Registry	9069	PDAC	*62.1*	27	44.8	56.9 Neoadjuvant CTx: 3.2	30-day: 2.6	–	33.2	–	5.5	
	Norway 2003–2014 Cancer Registry of Norway	6178	PDAC	*75*	10.3	20.6	24 No neoadjuvant data	30-day: 0.75	–	20.2	–	3.4	
	Slovenia 2003–2013 Cancer Registry of Slovenia	2664	PDAC	*75*	20.3	12.4	29.4 Neoadjuvant CTx: 0.5	30-day: 1.3	–	22.8	–	3.3	
Bengtsson (2020)^1^	USA 2004–2011 SEER	38 708	PDAC	57.4	19.5	54.4 for the whole population	–	–	–	–	–	3.8	M
Creighton (2017)^22^	Australia 2005–2009 Admitted Patient, ED, Attendance and Deaths Register	3473	Pancreatic cancer, excluded NET and ‘rare histological types’	46.5	11.6	–	–	90-day: 6.5	–	–	–	5.1	M
Latenstein (2020)^37^	Netherlands 1997–2016 Netherlands Cancer Registry	36 453	PDAC	52.4	12	15.6	37.5 Neoadjuvant CTx: 3.8	–	3.5	–	–	–	M
	Netherlands 1997–2000 As above	5572	PDAC	45.2	8.3	7.4	3 Neoadjuvant CTx: 0.2	90-day: 10.5	3.1	13.4	2.3	1.3	
	Netherlands 2001–2004 As above	5858	PDAC	–	8.3	9.7	6.8 Neoadjuvant CTx: 0.8	90-day: 11.1	3.2	14.1	2.8	1.2	
	Netherlands 2005–2008 As above	7179	PDAC	–	10.2	13.7	21.1 Neoadjuvant CTx: 1	90-day: 6.4	3.5	14.7	3	1.6	
	Netherlands 2009–2012 As above	8470	PDAC	–	13.6	19.5	49.5 Neoadjuvant CTx: 2.1	90-day: 6.9	3.7	18.5	4.6	2.5	
	Netherlands 2013–2016 As above	9374	PDAC	57	16.6	22	56.2 Neoadjuvant CTx: 8.5	90-day: 5	3.8	21	5.4	3.4	
Nymo (2022)^43^	Norway 2004–2008 Cancer Registry of Norway	3287	PDAC	–	10.8	–	–	90-day: 3.2	3.7	17.8	–	–	M
	Norway 2014–2018 Cancer Registry of Norway	3925	PDAC	–	17	For 2010–2018: 45	For 2010–2018: 68.1		5.8	29.9	–	–	
Whitley (2023)^42^	Czech 1985–2015 National Oncological registry of the Czech Republic	18 888	PDAC	39.7	17.2	27.3 for the whole population	–	–	–	23	6.9	4.4	S
Pilgrim (2023)^33^	Australia 2011–2015 Crosslinkage of Victorian Cancer Registry, Victorian Admitted Episodes Data set, Victorian Radotherapy Minimum Data Set, Victorian Emergency Minimum Data set and Victorian Death Index	3293	PDAC	67	12.9	Non-metastatic pts: 32 Metastatic pts: 44	Non-metastatic pts: 77 Neoadjuvant CTx: 4	–	–	29.7	–	–	M
	Australia 2016–2019 As above	3138	PDAC	63	14.2	Non-metastatic pts: 33 Metastatic pts: 43	Non-metastatic pts: 76 Neoadjuvant CTx : 16	–	–	32.5	–	–	
Niederhuber (1995)^32^	USA 1985/86 + 1991 ACSNCDB	7882	Pancreatic cancer	50.7	14.2	75.2	25.3	–	–	25	7	4	S
Sener (1999)^31^	USA 1985–1995 ACSNCDB	100 313	PDAC	33.4	9	32.8	40	–	–	–	–	6.1	S
Pasquali (2002)^29^	Italy 1990–1992 Regional database	253	Pancreatic cancer	–	17.7	–	–	In-hospital: 4.6	–	20.9	2.9	1.2	S
Keith (2022)^28^	Italy 2003–2011 Italy’s National Health Service administrative data	8158	Pancreatic cancer	36	20	40.3 for the whole population	–	–	6.4	–	–	–	M
Kagedan (2016)^30^	Canada 2005–2010 Ontario Cancer Registry	6296	PDAC	–	13	–	75.3	–	–	23.1†	7.5†	–	M

^*^CTx (chemotherapy) is adjuvant, or combination of adjuvant and neoadjuvant, if not specified. †Survival data from Cabasag *et al.* (survival of Canadian patients with pancreatic cancer excluding NETs 2012–2014)^44^. ROBINS-I, bias assessment; M, moderate; S, serious; PDAC, pancreatic ductal adenocarcinoma; NET, neuroendocrine tumour; SEER, Surveillance, Epidemiology, and End Results program; ACSNCDB, American College of Surgeons National Cancer Data Base; ED, emergency department. Huang15 “metastatic rate” italicised as study reported rate of stage 3 and 4 together.

A total of 516 789 patients were included, with a mean metastatic cancer rate of 54.2%. One study did not report a metastatic rate, instead combining stage 3 and 4 patients into one category (62.1%)^15^. The lowest population-level resection rate was 6% in Denmark from 1996 to 2003^41^, and the highest was 27% in Belgium from 2004 to 2013^15^. The pooled mean population resection rate was 14% (95% c.i. 12 to 15; *Fig. S2a*).

Eight studies reported perioperative mortality rate as in-hospital mortality rate (*n* = 1), 30-day mortality rate (*n* = 4) and/or 90-day mortality rate (*n* = 5) postresection. The studies varied in how they reported survival, including median OS (*n* = 9), and 1-year (*n* = 8), 3-year (*n* = 5) and 5-year (*n* = 12) survival. One study analysed patients with PDAC in the Ontario Cancer Register from 2005 to 2010 but did not report OS^30^. For that cohort, survival data was sourced from a different study that reported the survival of pancreatic cancer excluding NETs in 2012–2014^44^.

**Fig. 1:**
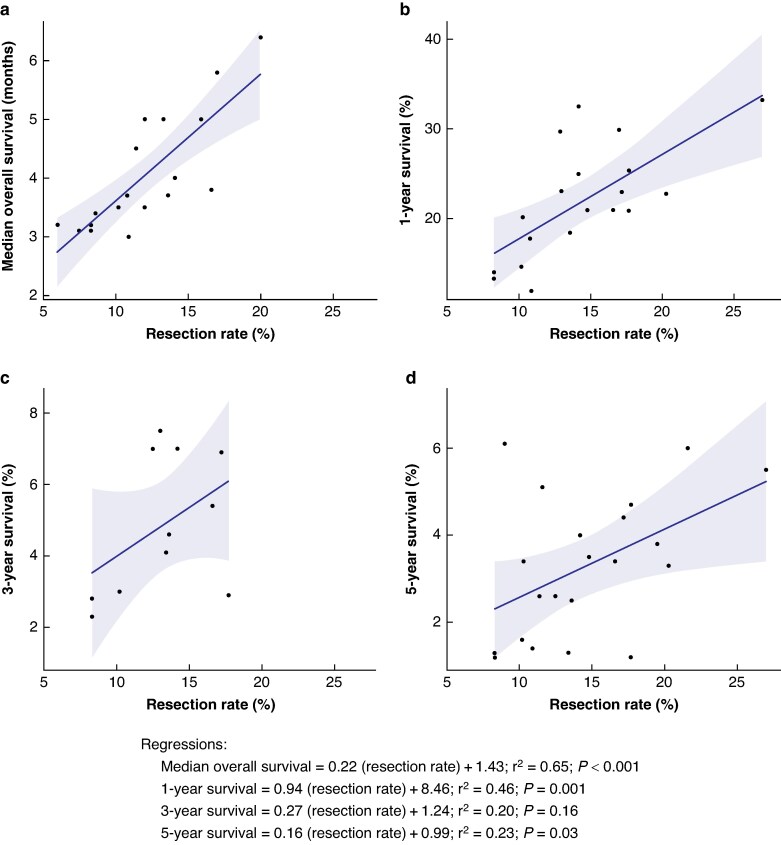
Survival *versus* resection rate: a median, b 1-year, c 3-year, d 5-year survival

Higher resection rates were significantly associated with increased median OS (r^2^ = 0.65, *P* < 0.001; *Fig. 1a*) and 1-year survival (r^2^ = 0.46, *P* = 0.001; *Fig. 1b*) at the population level. The association between resection rate and 3-year survival was weak and not significant (r^2^ = 0.20, *P* = 0.163; *Fig. 1c*), while the association between resection rate and 5-year survival was statistically significant but weak (r^2^ = 0.23, *P* = 0.029; *Fig. 1d*). The highest resection rate was 27% in the Belgian Cancer Registry in 2004–2013^15^, with 1- and 5-year survival rates of 33.2% and 5.5% respectively. This study only included PDAC and did not report a metastatic rate but had a combined stage 3 and 4 of 62.1%. The lowest resection rate was 6% in Denmark in 1996–2003^41^, with a metastatic rate of 37% and median OS of 32 months, which is in the lowest tertile of the studies reporting median OS.

**Fig. 2:**
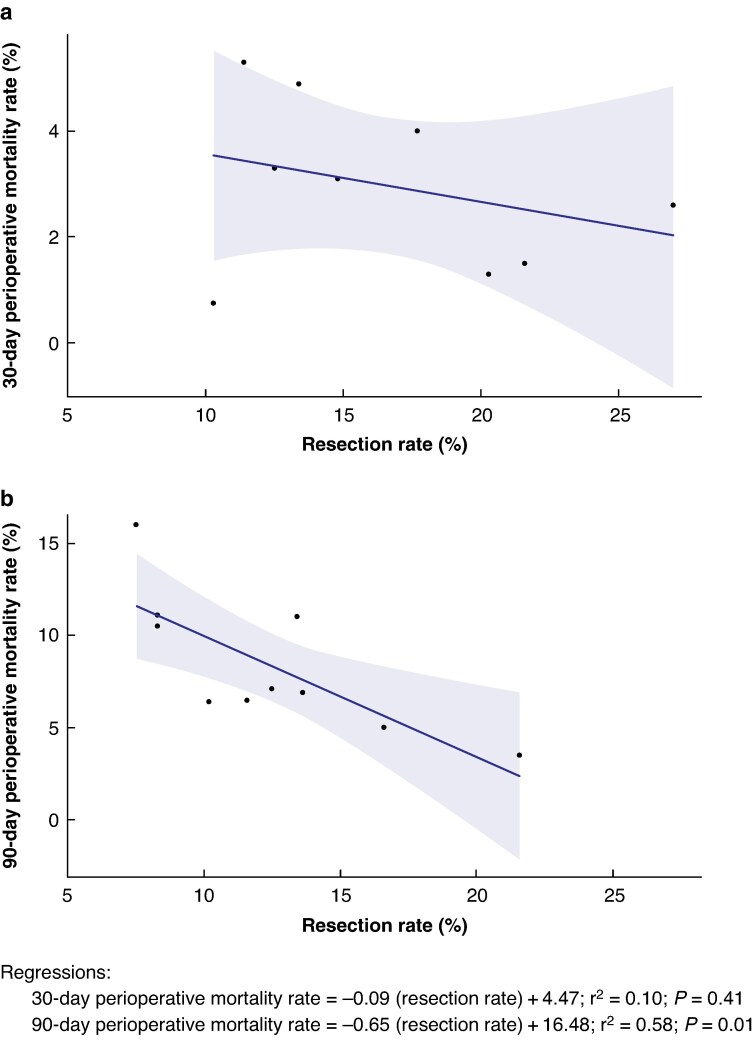
Perioperative mortality rate *versus* resection rate: a 30-day and b 90-day mortality rate

The in-hospital mortality rate was reported in a single study^29^, at 4.6% with a resection rate of 17.7%. The 30-day mortality rate ranged from 0.75% (Norwegian data^15^) to 4.9% (USA data^38^). At the highest resection rate of 27%, Belgian data^15^ reported a 30-day perioperative mortality rate of 2.6%. The 90-day mortality rate ranged from 3.5%^43^ to 16%^40,43^. The highest 90-day mortality rate of 16% also had the lowest resection rate (7.5%) in the studies that reported postoperative mortality rate. There was no association between resection rate and 30-day perioperative mortality rate (r^2^ = 0.10, *P* = 0.411; *Fig. 2a*), but there was a significant negative association between resection rate and 90-day perioperative mortality rate (r^2^ = 0.58, *P* = 0.007; *Fig. 2b*).

**Fig. 3:**
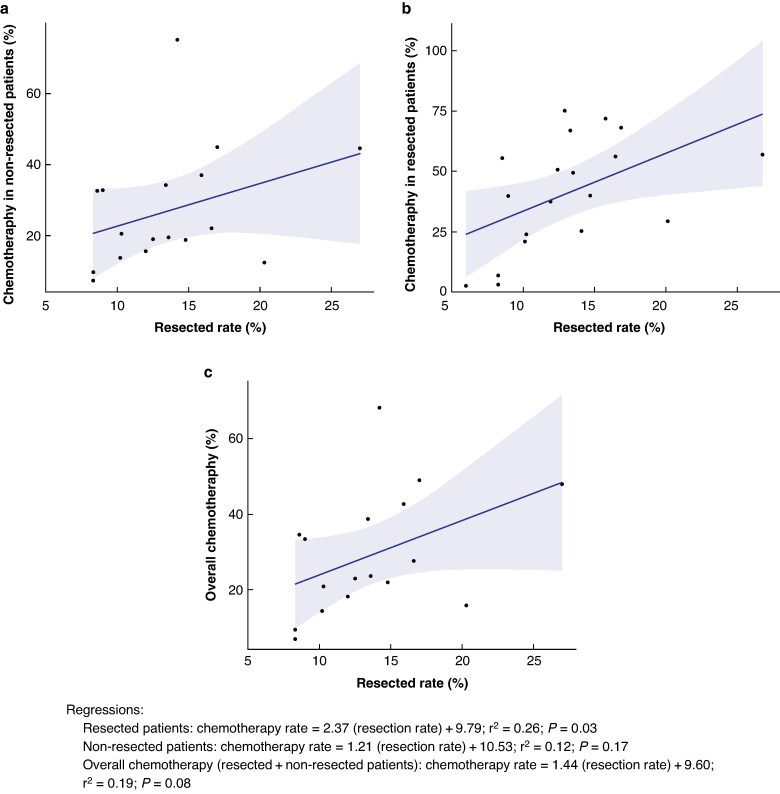
Chemotherapy

Twelve studies reported rates of adjuvant chemotherapy. Nine of these reported chemotherapy rates in the resected cohort, with three separating neoadjuvant from adjuvant therapy. In studies where neoadjuvant and adjuvant chemotherapy was not differentiated, the chemotherapy rate for resected patients was recorded as an overall rate of (neo)adjuvant chemotherapy. The (neo)adjuvant chemotherapy rates in resected patients ranged from 2.6 to 77%. Apart from one study^30^, these studies also reported palliative chemotherapy rates in non-resected patients, and this ranged from 7% (study period 1997–2000)^37^ to 75% (study period 1985/1986 and 1991)^32^. Three studies reported an overall chemotherapy rate for the whole-study population, not distinguishing between (neo)adjuvant and palliative intent. *Figure 3a* and *b* show chemotherapy rates in resected and non-resected patients relative to resection rate. There was a weak but significant association between resection rate and (neo)adjuvant chemotherapy in resected patients (r^2^ = 0.26, *P* = 0.027) but no association between the resection rate and palliative chemotherapy in the non-resected group (r^2^ = 0.12, *P* = 0.171). The relationship between the overall chemotherapy rate *versus* resection rate was not significant (r^2^ = 0.19, *P* = 0.082; *Fig. 3c*). The overall chemotherapy rate was associated with median OS (r^2^ = 0.76; *P* = 0.002) and survival at 1 year (r^2^ = 0.63; *P* = 0.004) but not at 3 or 5 years. For 1-year survival, the slope of the regression between survival and the overall chemotherapy rate was 0.26, compared with 0.94 for resection rate *versus* survival, suggesting that resection had a greater influence over survival than overall chemotherapy. The pooled mean population overall chemotherapy rate was 29% (95% c.i. 22 to 37; *Fig. S2b*).

**a** Chemotherapy in non-resected patients *versus* resection rate. **b** Chemotherapy in resected patients *versus* resection rate. **c** Overall chemotherapy *versus* resection rate.

*Figure S3a* and *3b* illustrates trends in the resection rate and chemotherapy rate over time. However, time intervals were non-uniform, preventing the fitting of regression lines. To further explore possible trends, a subset analysis of studies with data range of less than 5 years was conducted, as this may give a better indication of patterns in treatment over time. *Table S1* shows these studies, demonstrating there is an improvement in 1-year survival over time, from 13.4% in a study from 1997 to 2000 to 32.5% in a study from 2016 to 2019^33^. There is also an increasing trend in both resection rate and rate of chemotherapy in non-resected and resected patients. For example, the Latenstein^37^ study, which presents data from The Netherlands Cancer Registry in 4-year intervals from 1997 to 2016, shows a doubling of resection rate (8.3% to 16.6%), and a 197% increase in chemotherapy in non-resected patients (7.4 to 22%) and 1773% increase in resected patients (3% to 56.2%) from the earliest (1997–2000) to latest (2013–2016) time intervals. *Figure S4* demonstrates the trends in treatment regimens and 1-year survival over time in this subset of studies that also reported chemotherapy rates (Latenstein^37^ and Nymo^43^), demonstrating the improvement in survival and the increase in (neo)adjuvant chemotherapy and resection rate.

**Fig. 4:**
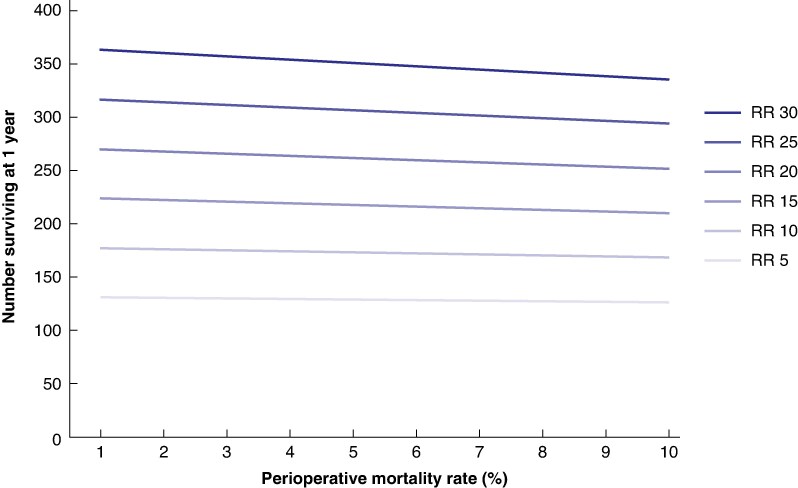
Model of resection rate and perioperative mortality rate for 1-year survival of a population of 1000 patients with pancreatic cancer

The number of survivors at 1 year from a population of 1000 patients with pancreatic cancer was modelled for different resection rates and perioperative mortality rate. For example, among 1000 patients with pancreatic cancer in a population with a resection rate of 10% and perioperative mortality rate of 2%, 100 will undergo resection, and 2 resected patients will die within 90 days. Using the regression line from data for 1-year survival *versus* resection rate, following a resection rate of 10%, the model predicted 176 patients from the initial 1000 population would survive to 1 year (17.6%). If resection rate increased to 15% and perioperative mortality rate to 3%, according to the model, this would lead to 221 patients from the 1000 population surviving to 1 year (22.1%). *Figure 4* demonstrates the model, indicating that 1-year survival improves with increasing resection rates, even if a higher perioperative mortality rate occurred. A similar pattern was found for 5-year survival (*Fig. S5*), although the number of survivors is evidently much lower.

RR, resection rate (%).

Assessment of bias is shown in *Table 1*, with full details in *Table S2*. Thirteen studies were assessed as moderate and six as serious risk of bias. Given all studies were observational studies, domain one for possible confounders was at least moderate for all. Eleven studies were restricted to analysis of patients with PDAC, the most common and most lethal type of pancreatic cancer^45^. For the four studies that did not specify beyond ‘pancreatic cancer’, the resection rate and survival may be skewed by other types of pancreatic cancer with better survival, including NETs^46^. The remaining types of pancreatic cancer are very rare, such that results from the remaining three studies that included pancreatic cancer but excluded NETs should not have been greatly influenced by other cancer types. The proportion of metastatic cases within the study cohort may also have influenced results, with two studies not reporting a metastatic rate, one reporting it only for some time intervals, one reporting stage 3 and 4 together, and three studies having unexpectedly low metastatic rates at 33.4%, 36% and 36.7%.

## Discussion

This review demonstrated that higher resection rates are associated with increased median, 1-year and 5-year survival in pancreatic cancer populations, including both resected and non-resected patients. There was a weak but significant association between resection rate and (neo)adjuvant chemotherapy in resected patients, although this association did not exist for palliative chemotherapy for non-resected patients. There was a significant negative association between resection rate and 90-day perioperative mortality rate. Our model predicted that a higher resection rate would result in more patients being alive at 1 and 5 years at a population level.

It is widely understood that resection of the primary pancreatic cancer improves survival at an individual level, when disease and patient characteristics are favourable for resection^6,7^. This may be due to removal of the tumour burden or prevention of complications from locoregional tumour progression (predominantly jaundice, cholangitis and duodenal obstruction). Autopsy studies have shown that up to one-third of patients with pancreatic cancer die from locoregional complications^47,48^. Concordantly, locoregional disease control with radiotherapy for unresectable pancreatic cancer was reported to be associated with a low mortality rate (7.1%) due to reduced locoregional complications^49^. The observation in our study that resection rates have a much stronger association with survival at 1 year compared with 3 or 5 years supports the hypothesis that locoregional control with resection may be a mechanism that improves survival. Additionally, patients who undergo resection, either because they are diagnosed earlier in the disease process or because they respond to neoadjuvant therapy, may have less aggressive tumour biology and thus have better survival^50–52^. Importantly, this study adds a novel perspective on the influence of resection rate on survival at a population level.

The mechanism by which the resection rate improves survival at a population level is likely multifactorial. Evidently, with increasing resection rates, more individuals will receive the survival benefit of resection and therefore increase the population survival. More importantly, healthcare systems that attain a higher resection rate likely achieve this through improving the performance in other aspects of the care continuum, thereby expanding the surgical pool. First, increased access to specialized care through overcoming barriers (for example socioeconomic, geographic, language, health literacy, etc.) may reduce the number of individuals who do not undergo surgery without an identifiable disease- or patient-specific contraindication, and thus improve the outcomes of previously marginalized populations. Similarly, better access may improve the time from presentation to specialist assessment, thereby accelerating earlier diagnosis and treatment. Expanding the pool of patients appropriate for surgery depends on the use of neoadjuvant therapy to downstage borderline resectable and locally advanced disease, perioperative optimization of co-morbidities, operating on higher risk patients in the context of an appropriate safety net that facilitates rescue following postoperative complications, and the use of advanced resection techniques for vascular and multivisceral resections. These levers can be manipulated at a population level and would increase the pool of surgical candidates. For example, Denmark had the lowest resection rate in the study before the introduction of a national integrated cancer patient pathway in 2007, which led to centralization of pancreatic surgery and an increase in annual pancreatic resection volumes^53^. Thus, the improved survival with higher resection rate is likely a surrogate marker for successful modification of these mechanisms in high-performing healthcare systems. Patients who do not undergo resection also benefit from improved care, such as higher chemotherapy rates, contributing to the improved survival at a population level. Certainly, there is a ceiling for resection where poor performance status or biologically high-risk disease would render surgery inappropriate. Both benchmarks and a ceiling for the resection rate currently remain undefined, although one review suggested a benchmark of 44% resection rate for pancreatic cancer in Australia^54^.

Increased use of neoadjuvant chemotherapy could render more disease resectable. This review showed that higher overall chemotherapy rates, particularly in surgical cohorts, are associated with improved survival. The study demonstrated an increase in overall chemotherapy as resection rate increased, which appeared to be driven by more (neo)adjuvant therapy. This suggests a potential ceiling in palliative chemotherapy use that is likely related to age, co-morbidities and performance status, and not significantly by other healthcare system factors. (Neo)adjuvant chemotherapy improved 1-year survival but had a weaker effect than resection rate. Thus, the improvement in survival with higher resection rate was not explained only by the increased use of chemotherapy, although it did have a role.

This study has demonstrated the existence of high-performance healthcare systems with high rates of resection and chemotherapy, as well as low perioperative mortality rates. There may be a concern that increasing resection rates could increase perioperative mortality rates, given the high risks of pancreatic surgery, which is why our model included increasing the perioperative mortality rate. However, this prediction is balanced by findings that high-volume centres, where increasingly radical resections are likely to occur, have higher rates of rescue due to better management of complications^55,56^. Even so, the survival models for 1 and 5 years demonstrated that a higher resection rate improves survival at the disease population level, even if there was an increased perioperative mortality rate of up to 10%, which is approximately three times the current benchmark^57^. This highlights that both perioperative mortality and resection rates need to be monitored, in order to improve survival for the pancreatic cancer population.

This study had several limitations. Survival data were heterogeneously reported, necessitating separate analysis for different time points of survival. Another limitation is that data in this review covers a time that saw the introduction of more effective chemotherapy regimens, including gemcitabine (with or without nano-albumin-bound paclitaxel), FOLFIRINOX (fluorouracil leucovorin irinotecan oxaliplatin) and S-1^58,59^, which occurred around 2011 with only three studies being after this. Additionally, there were paradigm shifts in the use of neoadjuvant chemotherapy for borderline resectable and locally advanced cancer, and there was insufficient detail in the studies regarding chemotherapy regimens used^7^. Therefore, it was not possible to perform a subgroup analysis of studies restricted to new chemotherapy era or agents. There was heterogeneity in the time interval for each study, ranging from 3 to 40 years (median 7), which prevented an analysis of the relationship between resection rate and year. Additionally, in each study the resection rate was presented as an average for the entire study interval, although it may have varied over time. Subset analysis of studies with shorter date ranges revealed an increasing trend in resection rate, chemotherapy rate and 1-year survival. This reinforces that the improvement in survival is associated with both increasing resection and chemotherapy, however, the regression analysis has shown that the effect of resection on survival at 1 year was stronger than the effect of chemotherapy. The analysis was also limited by relatively few studies that reported the perioperative mortality rate, and that the per capita resection rate could not be accurately calculated due to the unknown denominator of the overall population. Regarding the risk of bias assessment in which 13 studies had moderate and 6 had serious bias, this is in the context of the ROBINS-I tool equating low risk with a well-performed randomized trial and moderate risk being considered ‘sound for a non-randomized study’^27^.

This review has demonstrated that an increasing resection rate for pancreatic cancer is associated with improved survival at a disease population level. While there are recognized limitations, this study represents the best available data for this relationship. Therefore, to improve the short- and medium-term survival for pancreatic cancer, addressing healthcare systems that increase the resection rate is a priority. This could be achieved through earlier diagnosis, reducing barriers in access to healthcare, expanding the surgical population through use of downstaging neoadjuvant therapies, increasing the radicality of surgery, improving the efficacy of preoperative optimization and operating on higher risk patients in the context of a safety net that facilitates rescue following postoperative complications. Together, these mechanisms could improve survival for the entire pancreatic cancer disease population, even if this were to result in a modestly higher perioperative mortality rate. Evidently, there is a ceiling for resection where poor performance status or biologically high-risk disease would render surgery inappropriate. There is currently no benchmark for a pancreatic cancer resection rate, but the first step should be for regions to determine their resection rate and identify safe and appropriate ways to increase it. Establishing a benchmark resection rate would guide equitable access to high-quality pancreatic cancer care without promoting medically futile treatments.

## Supplementary Material

zraf007_Supplementary_Data

## Data Availability

The authors confirm that the data supporting the findings of this study are available within the article and its *Supplementary material*.
