# Supplementary material for: Relationship between pancreatic cancer resection rate and survival at population level: systematic review
Source: BJS Open. 2025 Mar 25;9(2):zraf007. doi: 10.1093/bjsopen/zraf007 (PMC11934921; doi:10.1093/bjsopen/zraf007)
Supplement: zraf007_Supplementary_Data [file zraf007_supplementary_data.zip › Supplementary_Material.docx]

**The relationship between pancreatic cancer resection rate and survival at population level: systematic review**

Dr Elizabeth B Lockie ^1,2^ BBiomed MStat MD

Dr Amy Sylivris^1^ MD

Professor Sanjay Pandanaboyana^3,4^ MS MPhil FRCS

Professor John Zalcberg AO^1,5,6^ MBBS PhD FRACP FRACMA FAHMS FAICD

Associate Professor Anita Skandarajah^1,2,7^ MBBS MD FRACS FACS

Associate Professor Benjamin P Loveday^1,2,7^ MBChB PhD FRACS

^1^The University of Melbourne

^2^Royal Melbourne Hospital

^3^Freeman Hospital, Newcastle upon Tyne, UK

^4^Population Health Sciences Institute, Newcastle University, Newcastle upon Tyne, UK

^5^School of Public Health, Faculty of Medicine, Monash University

^6^Dept. of Medical Oncology, Alfred Health

^7^Peter MacCallum Cancer Centre

**Corresponding author (& author for reprints)**:

Elizabeth Lockie

[Elockie18@gmail.com](mailto:Elockie18@gmail.com)

+61 448 546 906

Department of General Surgical Specialties, Royal Melbourne Hospital, 300 Grattan St, Parkville, Australia 3052

ORCHID ID: 0000-0002-5223-5958

**Supplementary Materials - Index**

| **Supplementary Methods** |  |
| --- | --- |
| Full search terms | *page 3* |
| **Supplementary Figures and Tables** |  |
| Supplemental Table 1. Subset of studies with date range of less than five years: resection rate, chemotherapy rates, and survival | *page 4* |
| Supplemental Table 2. ROBINS-I risk of bias assessment | *page 5* |
| Supplemental Figure 1. Flow chart of literature review | *page 7* |
| Supplemental Figure 2. Forest plot of single arm meta-analysis of  2A. Forest plot of resection rate  2B. Forest plot of overall chemotherapy rate | *page 8* |
| Supplemental Figure 3. Patterns in treatment over time (all studies)  3A. Resection and overall chemotherapy rates over time  3B. Chemotherapy rates in non-resected and resected patients over time | *page 9* |
| Supplemental Figure 4. Patterns in treatment and survival over time, in subset of studies with date range less than five years | *page 10* |
| Supplemental Figure 5. Model of resection rate and perioperative mortality for five-year survival of a population of 1000 pancreatic cancer patients | *page 11* |
| **References** | *page 12* |

**Supplementary Methods**

**Full search terms**

Ovid MEDLINE full search criteria (1946 to February 20, 2024)

1 (pancrea* adj3 (cancer* or neoplasm* or tumour* or tumor* or malignan* or adenocarcinoma)).tw.

2 Pancreatectomy/ or Pancreaticoduodenectomy/

3 Survival Rate/ or Survival/

4 Mortality/

5 3 or 4

6 Pancreatic Neoplasms/mo, su [Mortality, Surgery]

7 1 or 6

8 2 and 5 and 7

9 limit 8 to english language

Evidence Based Medicine Reviews (Cochrane Central Register of Controlled trials) full search criteria (January 2024)

1 (pancrea* adj3 (cancer* or neoplasm* or tumour* or tumor* or malignan* or adenocarcinoma)).tw.

2 Pancreatectomy/ or Pancreaticoduodenectomy/

3 Survival Rate/ or Survival/

4 Mortality/

5 3 or 4

6 Pancreatic Neoplasms/

7 1 or 6

8 2 and 5 and 7

9 limit 8 to english language

Embase full search criteria (1947 to 2024 February 20)

1 (pancrea* adj3 (cancer* or neoplasm* or tumour* or tumor* or malignan* or adenocarcinoma)).tw.

2 Pancreatectomy/ or Pancreaticoduodenectomy/

3 Survival Rate/ or Survival/

4 Mortality/

5 3 or 4

6 Pancreatic Neoplasms/mo, su [Mortality, Surgery]

7 1 or 6

8 2 and 5 and 7

9 limit 8 to english language

**Supplementary Figures and Tables**

**Supplemental Table 1. Subset of studies with date range of less than five years: resection rate, chemotherapy rates, and survival**

|  |  |  | Chemotherapy | | Survival | | | |
| --- | --- | --- | --- | --- | --- | --- | --- | --- |
| **Study** | **Study years** | **Resection rate (%)** | **Non-resected patients** | **Resected patients** | **Median survival (months)** | **1-year (%)** | **3-year (%)** | **5-year (%)** |
| Wakeman  2004 ^42^ | 1994-1997 | 7.5 | NA | NA | 3.1 |  |  |  |
| Speer 2012 ^41^ | 2002-2003 | 11.4 | NA | NA | 4.5 |  |  | 2.6 |
| Creighton 2017 ^23^ | 2005-2009 | 11.6 | NA | NA |  |  |  | 5.1 |
| Latenstein 2020 ^39^ | 1997-2000 | 8.3 | 7.4 | 3 | 3.1 | 13.4 | 2.3 | 1.3 |
|  | 2001-2004 | 8.3 | 9.7 | 6.8 | 3.2 | 14.1 | 2.8 | 1.2 |
|  | 2005-2008 | 10.2 | 13.7 | 21.1 | 3.5 | 14.7 | 3 | 1.6 |
|  | 2009-2012 | 13.6 | 19.5 | 49.5 | 3.7 | 18.5 | 4.6 | 2.5 |
|  | 2013-2016 | 16.6 | 22 | 56.2 | 3.8 | 21 | 5.4 | 3.4 |
| Nymo 2022 ^34^ | 2004-2008 | 10.8 | NA | NA | 3.7 | 17.8 |  |  |
|  | 2014-2018 | 17 | 45 | 68.1 | 5.8 | 29.9 |  |  |
| Pilgrim  2023 ^35^ | 2011-2015 | 12.9 | NA | NA |  | 29.7 |  |  |
|  | 2016-2019 | 14.2 | NA | NA |  | 32.5 |  |  |
| Niederhuber 1995 ^33^ | 1985/86 + 1991 | 14.2 | 75.2 | 25.3 |  | 25 | 7 | 4 |
| Pasquali 2002 ^30^ | 1990-1992 | 17.7 | NA | NA |  | 20.9 | 2.9 | 1.2 |

## Studies highlighted in red – used to construct Supplemental Figure 4 as continuous time periods and reported chemotherapy rates

## Supplemental Table 2. ROBINS-I risk of bias assessment

| **Study ID** | **Bias due to confounding*** | **Bias due to selection of participants** | **Bias in classification of interventions** | **Bias due to missing data** | **Overall** |
| --- | --- | --- | --- | --- | --- |
| Tingstedt 2019 ^36^ | Did not specify metastatic rate (">50%"). Did not include chemotherapy rates; states due to poor collection in the registry of this information. | PDAC only | Resection included pancreatoduodenectomy (PD), total pancreatectomy (TP) and distal pancreatectomy (DP) | States prior to 2012 there was "low coverage" but since 2012 >85% coverage. Does not comment on loss to follow up |  |
|  | Serious | Low | Moderate | Moderate | Moderate |
| Salami 2019 ^37^ | Did not include chemotherapy rates, states this is due to inaccuracy in the SEER database about this | PDAC only | Does not state if resection meant only PD | SEER covers 48% of the US population. |  |
|  | Serious | Moderate | Moderate | Serious | Moderate |
| Linder 2007 ^38^ | No metastatic rate reported | PDAC only | Does not state if resection meant only PD | Data linkage study (states study shown <1% of patients in the database have incorrect diagnosis), linked with national deaths database |  |
|  | Serious | Low | Moderate | Moderate | Moderate |
| Kirkegard 2022 ^43^ | The 1996-2203 cohort had low metastatic rate (36%) compared to the norm | 68% PDAC | Resection included PD, TP and DP, however low volume TP and DP | Data linkage study, linked with national deaths database |  |
|  | Serious | Moderate | Moderate | Moderate | Moderate |
| Wakeman 2004 ^42^ | No metastatic rate reported. No chemotherapy rates reported | Included all "pancreatic cancer" | PD only | Data linkage study, linked with national deaths database |  |
|  | Serious | Serious | Low | Moderate | Serious |
| Speer 2012 ^41^ | No metastatic rate reported. No chemotherapy rates reported | Pancreatic cancer, excluded NET and ampulla of Vater tumours | PD only | Data linkage study, linked with national deaths database |  |
|  | Serious | Serious | Low | Moderate | Moderate |
| Saadat 2024 ^40^ | No metastatic rate reported | Pancreatic cancer, excluded NET | Resection included PD, DP and other pancreatectomy | Ontario cancer registry includes linked administrative data for all cancer patients in Ontario. SEER covers 48% of the US population; excluded US patients if did not have 12 months of follow up (or death). |  |
|  | Serious | Serious | Moderate | Serious (due to SEER) | Serious |
| Huang 2018 ^15^ | No metastatic rate reported but reported stage 3/4 together. Excluded unstaged cancers. | PDAC | Resection included PD, TP and DP | Excluded national registries that were not able to demonstrate high quality data or if population too small for survival analysis (e.g. Estonia) |  |
|  | Moderate-serious | Moderate | Moderate | Moderate | Moderate |
| Bengtsson 2020 ^1^ | Chemotherapy rate for overall population only. | PDAC | Resection included PD, TP and DP | SEER covers 48% of the US population, but this study only included patients with a complete 5 years of follow up (or death prior) |  |
|  | Serious | Moderate | Moderate | Moderate | Moderate |
| Creigthon 2017 ^23^ | Adjusted for confounding factors | Pancreatic cancer, excluded NET and "rare histological types" | Resection included PD, TP and DP; but the analysis by surgery type was PD vs other | Data linkage study, linked with national deaths database |  |
|  | Moderate | Serious | Moderate | Moderate | Moderate |
| Latenstein 2020 ^39^ | Metastatic rate not reported for every period | PDAC | Resection included PD, TP and DP | Registries with data linkage |  |
|  | Serious | Moderate | Moderate | Moderate | Moderate |
| Nymo 2022 ^34^ | No metastatic rate reported. Only had chemotherapy rate for second time period as registry was incomplete for this prior to 2010 | PDAC | Resection included PD, TP and DP | The Registry is linked with the national deaths database |  |
|  | Serious | Moderate | Moderate | Moderate | Moderate |
| Whitley 2023 ^44^ | Chemotherapy rate for overall population only. | PDAC | Resection included PD, TP and DP | Does not state if linked to deaths registry |  |
|  | Serious | Moderate | Moderate | Serious | Serious |
| Pilgrim 2023 ^35^ | Reported metastatic rate and chemotherapy rate for stages | PDAC | Resection included PD, TP and DP; but PD outcomes were noted | Data linkage study, linked with national deaths database |  |
|  | Moderate | Moderate | Moderate | Moderate | Moderate |
| Niederhuber 1995 ^33^ | Reported metastatic rate and chemotherapy rates | Pancreatic cancer | Resection included PD, TP and DP | States the 1991 data includes 31% of pancreatic ca cases across US. Participation in the the NCDB is voluntary |  |
|  | Moderate | Serious | Moderate | Serious | Serious |
| Sener 1999 ^32^ | Low metastatic rate (33.4%) in voluntary registry may mean patients with less advanced cancer may have been reported to the registry | PDAC | Resection included PD, TP and DP | Participation in the the NCDB is voluntary |  |
|  | Serious | Moderate | Moderate | Serious | Serious |
| Pasquali 2022 ^30^ | No metastatic rate reported, no chemotherapy reported | Pancreatic cancer | Resection included PD, TP and DP | Abstract only so limited information re data collection process |  |
|  | Serious | Serious | Moderate | Serious | Serious |
| Keith 2002 ^29^ | Low metastatic rate, overall chemotherapy rate reported only, | Pancreatic cancer | Resection included PD, TP and DP | Data linkage study, linked with national deaths database |  |
|  | Serious | Serious | Moderate | Moderate | Moderate |
| Kagedan 2016 ^31^ | No metastatic rate reported | PDAC | Resection included PD, TP and DP | Ontario cancer registry includes linked administrative data for all cancer patients in Ontario. |  |
|  | Serious | Moderate | Moderate | Moderate | Moderate |

**D1: at least all moderate as registry-based data so cannot account for all confounders*

*Weight: all weighted as 1*

*Note that in the ROBINS-I tool for bias assessment, the interpretation of the judgement is as follows^28^:*

- *Low risk: The study is comparable to a well conducted randomised trial*
- *Moderate risk: The study is sound for a non-randomised trial*
- *Serious risk: The study has some serious problems*
- *Critical risk: The study is too problematic to provide any useful evidence*

**Supplemental Figure 1. Flow chart of the literature review**

Studies from databases/registers **(n = 4822)**

Embase (n = 3339)

MEDLINE (n = 1422)

EBMR (n = 58)

Other including citation search (n = 3)

**Identification**

**Screening**

Studies included in review **(n = 19)**

**Included**

Studies excluded **(n = 47)**

Wrong setting – single centre (n = 10)

Wrong outcomes – no overall survival (n = 16)

Wrong study design (n = 4)

Wrong patient population (n = 14)

Abstract only - insufficient results (n = 3)

Studies excluded **(n = 3901)**

References removed **(n = 855)**

Duplicates identified manually (n = 47)

Duplicates identified by Covidence (n = 808)

Marked as ineligible by automation tools (n = 0)

Studies assessed for eligibility **(n = 66)**

Studies sought for retrieval **(n = 66)**

Studies screened **(n = 3967)**

**Supplemental Figure 2. Forest plot of single arm meta-analysis**

Supplemental Figure 2A. Forest plot of resection rate


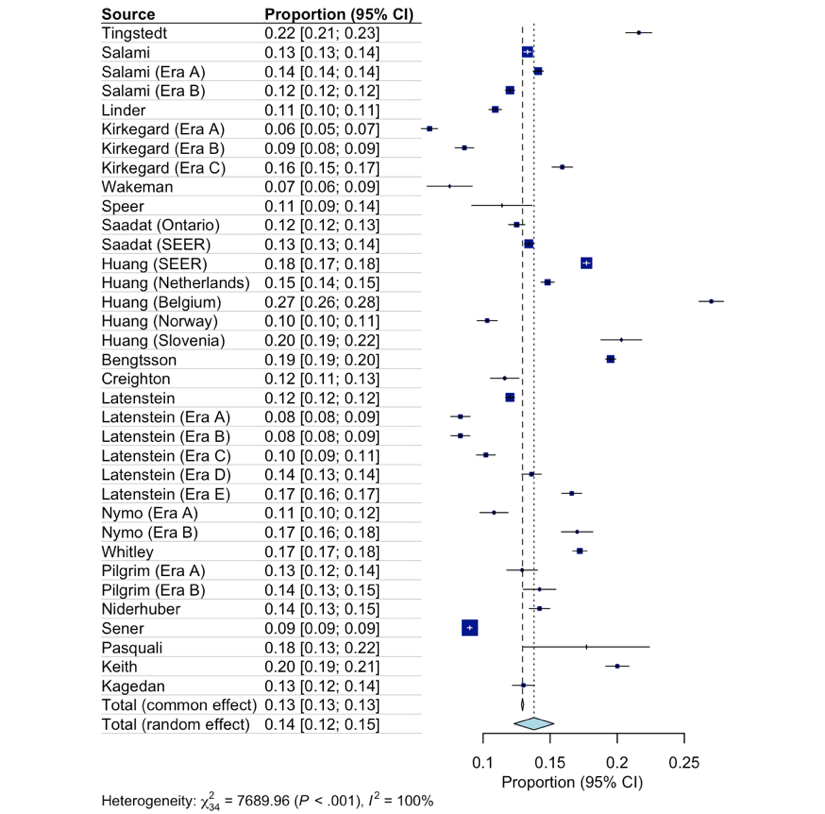


Supplemental Figure 2B. Forest plot of overall chemotherapy rate


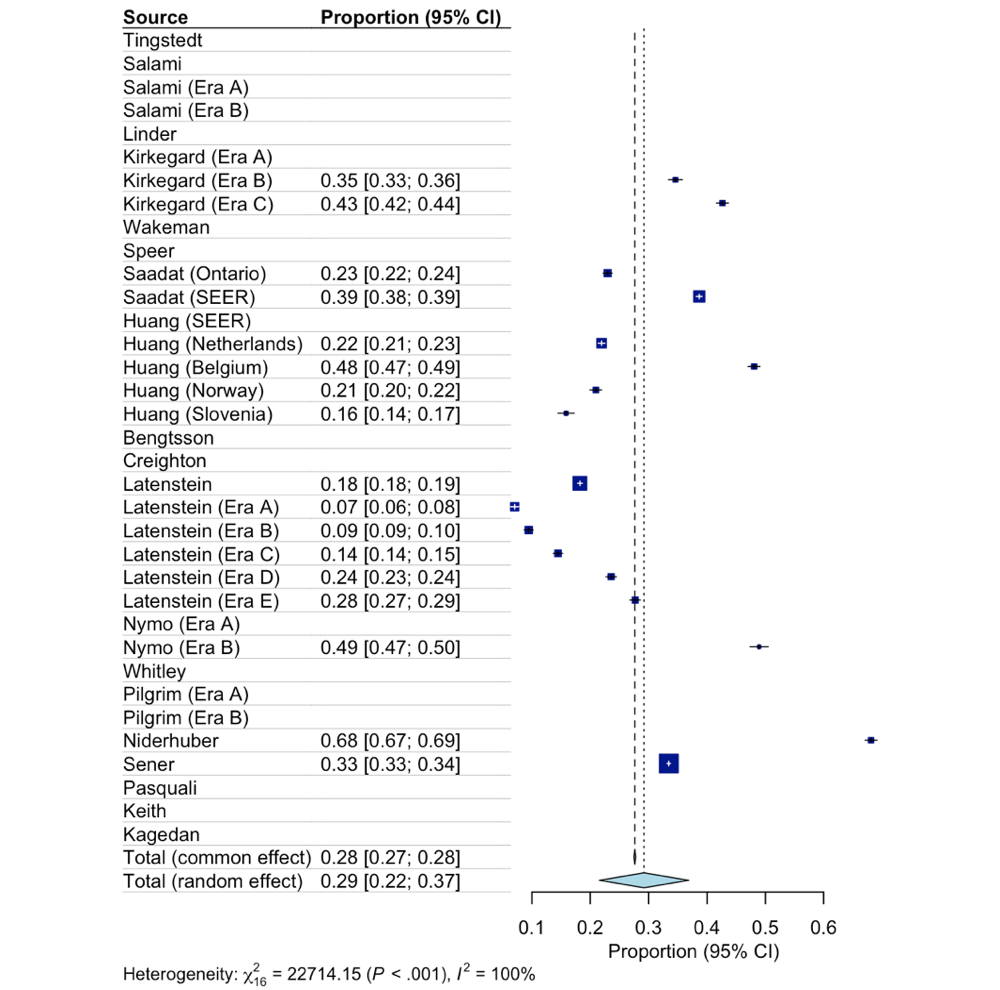


##

**Supplemental Figure 3. Patterns in treatment over time (all studies)**

Supplemental Figure 3A. Resection and overall chemotherapy* rates over time^

Supplemental Figure 3B. Chemotherapy rates in non-resected and resected patients over time^

CTx = chemotherapy

*Overall chemotherapy = chemotherapy in resected (neoadjuvant & adjuvant chemotherapy) and non-resected patients (palliative chemotherapy)

^Studies ordered over time by the first year of the study

**Supplemental Figure 4. Patterns in treatment and survival over time, in subset of studies with date range less than five years that reported chemotherapy**

Studies contributing to this graph – Latenstein^39^ and Nymo^3^

**Supplemental Figure 5. Model of resection rate and perioperative mortality for five-year survival of a population of 1000 pancreatic cancer patients**

Model based on regression from study’s data for five-year survival starting with a population of 1000 pancreatic cancer patients (operative and non-operative candidates)

Abbreviations: RR = resecation rate (%)

**References**

1 Bengtsson A, Andersson R, Ansari D. The actual 5-year survivors of pancreatic ductal adenocarcinoma based on real-world data. *Sci Rep*. Nature Research; 2020 Dec 1; **10**.

2 American Cancer Society. Cancer Facts and Figures 2024 [Internet]. Atlanta; 2024. Available from: American Cancer Society. Cancer Facts & Figures 2024. Atlanta: American Cancer Society; 2024.

3 Australian Institute of Health and Welfare. Cancer data in Australia [Internet]. 2023. Available from: https://www.aihw.gov.au/reports/cancer/cancer-data-in-australia

4 Pourshams A, Sepanlou SG, Ikuta KS, Bisignano C, Safiri S, Roshandel G, *et al.* The global, regional, and national burden of pancreatic cancer and its attributable risk factors in 195 countries and territories, 1990–2017: a systematic analysis for the Global Burden of Disease Study 2017. *Lancet Gastroenterol Hepatol*. Elsevier Ltd; 2019 Dec 1; **4**: 934–947.

5 Punjwani S, Jaroenlapnopparat A, Jani C, Singh H, Marshall DC, Salciccioli JD, *et al.* An estimate of the burden of pancreatic cancer globally and its comparison with different WHO regions using Global Burden of Disease Database: A retrospective population-based analysis. *Journal of Clinical Oncology* [Internet]. Wolters Kluwer; 2023 May 31; **41**: e16321–e16321. Available from: https://doi.org/10.1200/JCO.2023.41.16_suppl.e16321

6 Raoof S, Clarke CA, Hubbell E, Chang ET, Cusack J. Surgical resection as a predictor of cancer-specific survival by stage at diagnosis and cancer type, United States, 2006–2015. *Cancer Epidemiol*. Elsevier Ltd; 2023 Jun 1; **84**.

7 Tempero MA, Malafa MP, Al-Hawary M, Asbun H, Bain A, Behrman SW, *et al.* Pancreatic adenocarcinoma, version 2.2017: Clinical practice guidelines in Oncology. JNCCN Journal of the National Comprehensive Cancer Network. Harborside Press; 2017 Aug.

8 Pausch TM, Liu X, Cui J, Wei J, Miao Y, Heger U, *et al.* Survival benefit of resection surgery for pancreatic ductal adenocarcinoma with liver metastases: A propensity score-matched SEER database analysis. *Cancers (Basel)*. MDPI; 2022 Jan 1; **14**.

9 Brada LJH, Daamen LA, Magermans LG, Walma MS, Latifi D, van Dam RM, *et al.* Survival Benefit Associated With Resection of Locally Advanced Pancreatic Cancer After Upfront FOLFIRINOX Versus FOLFIRINOX Only Multicenter Propensity Score-matched Analysis. *Ann Surg*. Lippincott Williams and Wilkins; 2021 Nov 1; **274**: 729–735.

10 Hartwig W, Gluth A, Hinz U, Koliogiannis D, Strobel O, Hackert T, *et al.* Outcomes after extended pancreatectomy in patients with borderline resectable and locally advanced pancreatic cancer. *British Journal of Surgery*. John Wiley and Sons Ltd; 2016 Nov 1; **103**: 1683–1694.

11 Wang L, Yang L, Chen L, Chen Z. Do patients diagnosed with metastatic pancreatic cancer benefit from primary tumor surgery? A propensity-adjusted, population-based surveillance, epidemiology and end results (SEER) analysis. *Medical Science Monitor*. International Scientific Information, Inc.; 2019 Nov 2; **25**: 8230–8241.

12 Voss N, Izbicki JR, Nentwich MF. Oligometastases in pancreatic cancer (Synchronous resections of hepatic oligometastatic pancreatic cancer: Disputing a principle in a time of safe pancreatic operations in a retrospective multicenter analysis). *Ann Gastroenterol Surg*. Wiley-Blackwell Publishing Ltd; 2019 Jul 1; **3**: 373–377.

13 Ghaferi AA, Birkmeyer JD, Dimick JB. Complications, failure to rescue, and mortality with major inpatient surgery in medicare patients. *Ann Surg*. 2009 Dec; **250**: 1029–1033.

14 Hartwig W, Werner J, Jäger D, Debus J, Büchler MW. Review Improvement of surgical results for pancreatic cancer. *The Lancet Oncology* [Internet]. 2013; **14**: e476–e485. Available from: www.thelancet.com/oncology

15 Huang L, Jansen L, Balavarca Y, Babaei M, van der Geest L, Lemmens V, *et al.* Stratified survival of resected and overall pancreatic cancer patients in Europe and the USA in the early twenty-first century: A large, international population-based study. *BMC Med*. BioMed Central Ltd.; 2018 Aug 21; **16**.

16 Bilimoria KY, Bentrem DJ, Ko CY, Stewart AK, Winchester DP, Talamonti MS. National failure to operate on early stage pancreatic cancer. *Ann Surg*. 2007 Aug; **246**: 173–180.

17 Murimwa GZ, Karalis JD, Meier J, Nehrubabu M, Thornton M, Porembka M, *et al.* Factors associated with failure to operate and its impact on survival in early-stage pancreatic cancer. *J Surg Oncol*. John Wiley and Sons Inc; 2023 Sep 15; **128**: 540–548.

18 Ghaferi AA, Birkmeyer JD, Dimick JB. Complications, failure to rescue, and mortality with major inpatient surgery in medicare patients. *Ann Surg*. 2009 Dec; **250**: 1029–1033.

19 Powers BD, McDonald J, Mhaskar R, Lee SJC, Permuth JB, Vadaparampil S, *et al.* Hospital Surgical Volume Is Poorly Correlated With Delivery of Multimodal Treatment for Localized Pancreatic Cancer. *Annals of Surgery Open*. Ovid Technologies (Wolters Kluwer Health); 2022 Sep; **3**: e197.

20 Burmeister EA, O DL, Beesley VL, Goldstein D, Gooden HM, Janda M, *et al.* Describing Patterns of Care in Pancreatic Cancer A Population-Based Study. *Pancreas* [Internet]. 2015; **44**: 1259–1265. Available from: www.pancreasjournal.com

21 Shah A, Chao KSC, Østbye T, Castleberry AW, Pietrobon R, Gloor B, *et al.* Trends in Racial Disparities in Pancreatic Cancer Surgery. *Journal of Gastrointestinal Surgery*. 2013 Nov; **17**: 1897–1906.

22 Shapiro M, Chen Q, Huang Q, Boosalis VA, Yoon CH, Saund MS, *et al.* Associations of socioeconomic variables with resection, stage, and survival in patients with early-stage pancreatic cancer. *JAMA Surg*. American Medical Association; 2016 Apr 1; **151**: 338–345.

23 Creighton N, Walton R, Roder DM, Aranda S, Richardson AJ, Merrett N, *et al.* Pancreatectomy is underused in NSW regions with low institutional surgical volumes: a population data linkage study. *Medical Journal of Australia* [Internet]. 2017; **206**: 23–28. Available from: <Go to ISI>://WOS:000397098700009

24 Karin Johansen Thomas Gasslander Kristina Hasselgren Per Sandstrom Bergthor Bjornsson LL. High resection rate improves overall survival in elderly patients with pancreatic head cancer: A cohort study. *International Journal of Surgery Open*. 2021; **34**.

25 Grant SB, Modi PK, Singer EA. Futility and the care of surgical patients: Ethical dilemmas. *World J Surg*. Springer New York LLC; 2014; **38**: 1631–1637.

26 Shamseer L, Moher D, Clarke M, Ghersi D, Liberati A, Petticrew M, *et al.* Preferred reporting items for systematic review and meta-analysis protocols (PRISMA-P) 2015: Elaboration and explanation. *BMJ (Online)*. BMJ Publishing Group; 2015 Jan 2; **349**.

27 Covidence systematic review software [Internet]. Melbourne: Veritas Health Innovation; [cited 2024 Apr 27]. Available from: www.covidence.org

28 Sterne JA, Hernán MA, Reeves BC, Savović J, Berkman ND, Viswanathan M, *et al.* ROBINS-I: A tool for assessing risk of bias in non-randomised studies of interventions. *BMJ (Online)*. BMJ Publishing Group; 2016; **355**.

29 Keith SW, Maio V, Arafat HA, Alcusky M, Karagiannis T, Rabinowitz C, *et al.* Angiotensin blockade therapy and survival in pancreatic cancer: a population study. *BMC Cancer*. BioMed Central Ltd; 2022 Dec 1; **22**.

30 Pasquali C, Sperti C, Filipponi C, Pedrazzoli S. Epidemiology of pancreatic cancer in Northeastern Italy: incidence, resectability rate, hospital stay, costs and survival (1990–1992). *Digestive and Liver Disease*. 2002; **34**: 723–731.

31 Kagedan DJ, Abraham L, Goyert N, Li Q, Paszat LF, Kiss A, *et al.* Beyond the dollar: Influence of sociodemographic marginalization on surgical resection, adjuvant therapy, and survival in patients with pancreatic cancer. *Cancer*. John Wiley and Sons Inc.; 2016 Oct 15; **122**: 3175–3182.

32 Sener SF, Fremgen A, Menck HR, Winchester DP. Pancreatic Cancer: A Report of Treatment and Survival Trends for 100,313 Patients Diagnosed from 1985-1995, Using the National Cancer Database. *J Am Coll Surg* [Internet]. 1999; **189**: 1–7. Available from: http://journals.lww.com/journalacs

33 Niederhuber JE, Brennan MF, Menck HR. The national cancer data base report on pancreatic cancer. *Cancer*. 1995; **76**: 1671–1677.

34 Pilgrim CHC, Finn N, Stuart E, Philip J, Steel S, Croagh D, *et al.* Changing patterns of care for pancreas cancer in Victoria: the 2022 Pancreas Tumour Summit. *ANZ J Surg*. John Wiley and Sons Inc; 2023 Nov 1; **93**: 2638–2647.

35 Tingstedt B, Andersson B, Jönsson C, Formichov V, Bratlie SO, Öhman M, *et al.* First results from the Swedish National Pancreatic and Periampullary Cancer Registry. *HPB*. Elsevier B.V.; 2019 Jan 1; **21**: 34–42.

36 Salami A, Obaid T, Joshi ART. Trends in the clinical presentation, treatment, and survival for pancreatic adenocarcinoma. *Am J Surg*. Elsevier Inc.; 2019 Jan 1; **217**: 103–107.

37 Linder S, Boström L, Nilsson B. Pancreatic carcinoma incidence and survival in Sweden in 1980-2000: A population-based study of 16,758 hospitalized patients with special reference to different therapies. *European Journal of Surgical Oncology*. 2007 Jun; **33**: 616–622.

38 Latenstein AEJ, van der Geest LGM, Bonsing BA, Groot Koerkamp B, Haj Mohammad N, de Hingh IHJT, *et al.* Nationwide trends in incidence, treatment and survival of pancreatic ductal adenocarcinoma. *Eur J Cancer*. Elsevier Ltd; 2020 Jan 1; **125**: 83–93.

39 Saadat L V., Schofield E, Bai X, Curry M, Saskin R, Lipitz-Snyderman A, *et al.* Treatment Patterns and Outcomes in Pancreatic Cancer: A Comparative Analysis of Ontario and the USA. *Ann Surg Oncol*. Springer Science and Business Media Deutschland GmbH; 2024 Jan 1; **31**: 58–65.

40 Speer AG, Thursfield VJ, Torn-Broers Y, Jefford M. Pancreatic cancer: Surgical management and outcomes after 6 years of follow-up. *Medical Journal of Australia*. 2012 May; **196**.

41 Wakeman CJ, Martin IG, Robertson RW, Dobbs BR, Frizelle FA. Pancreatic cancer: Management and survival. *ANZ J Surg*. 2004 Nov; **74**: 941–944.

42 Kirkegård J, Bojesen AB, Nielsen MF, Mortensen FV. Trends in pancreatic cancer incidence, characteristics, and outcomes in Denmark 1980–2019: A nationwide cohort study. *Cancer Epidemiol*. Elsevier Ltd; 2022 Oct 1; **80**.

43 Whitley A, Kocián P, Nikov A, Krejčí D, Pehalová L, Blaha M, *et al.* Early-onset pancreatic cancer: A national cancer registry study from the Czech Republic and review of the literature. *J Hepatobiliary Pancreat Sci*. John Wiley and Sons Inc; 2023 Dec 1; **30**: 1324–1333.

44 Nymo LS, Myklebust TÅ, Hamre H, Møller B, Lassen K. Treatment and survival of patients with pancreatic ductal adenocarcinoma: 15-year national cohort. *BJS Open*. Oxford University Press; 2022 Apr 1; **6**.

45 Cabasag CJ, Arnold M, Rutherford M, Bardot A, Ferlay J, Morgan E, *et al.* Pancreatic cancer survival by stage and age in seven high-income countries (ICBP SURVMARK-2): a population-based study. *Br J Cancer*. Springer Nature; 2022 Jun 1; **126**: 1774–1782.

46 Park W, Chawla A, O’Reilly EM. Pancreatic Cancer: A Review. *J Am Med Assoc*. American Medical Association; 2021 Sep 7; **326**: 851–862.

47 Chang JS, Chen LT, Shan YS, Chu PY, Tsai CR, Tsai HJ. The incidence and survival of pancreatic cancer by histology, including rare subtypes: a nation-wide cancer registry-based study from Taiwan. *Cancer Med*. Blackwell Publishing Ltd; 2018 Nov 1; **7**: 5775–5788.

48 Lacobuzio-Donahue CA, Fu B, Yachida S, Luo M, Abe H, Henderson CM, *et al.* DPC4 gene status of the primary carcinoma correlates with patterns of failure in patients with pancreatic cancer. *Journal of Clinical Oncology*. 2009 Apr 10; **27**: 1806–1813.

49 Douglass HO, Penetrante RB. Pancreatic Cancer Why Patients Die. *International Journal of Pancreatology*. 1990; 135–140.

50 Chuong MD, Herrera R, Ucar A, Aparo S, De Zarraga F, Asbun H, *et al.* Causes of Death Among Patients With Initially Inoperable Pancreas Cancer After Induction Chemotherapy and Ablative 5-fraction Stereotactic Magnetic Resonance Image Guided Adaptive Radiation Therapy. *Adv Radiat Oncol*. Elsevier Inc.; 2023 Jan 1; **8**.

51 Truty MJ, Kendrick ML, Nagorney DM, Smoot RL, Cleary SP, Graham RP, *et al.* Factors Predicting Response, Perioperative Outcomes, and Survival Following Total Neoadjuvant Therapy for Borderline/Locally Advanced Pancreatic Cancer. *Ann Surg*. Wolters Kluwer Health; 2021 Feb 1; **273**: 341–349.

52 Yin L, Miao Y, Yu J. Advances of pathological complete response after neoadjuvant therapy for pancreatic cancer. *J Pancreatol*. Wolters Kluwer Health; 2019 Mar 1; **2**: 11–15.

53 Springfield C, Ferrone CR, Katz MHG, Philip PA, Hong TS, Hackert T, *et al.* Neoadjuvant therapy for pancreatic cancer. *Nat Rev Clin Oncol*. Springer Nature; 2023 May 1; **20**: 318–337.

54 Wennervaldt K, Kejs AM, Lipczak H, Bartels P, Borre M, Fristrup CW, *et al.* Regional variation in surgery for pancreatic cancer in Denmark 2011-2015. *Danish Medical Journal* . 2018; **65**.

55 Perera SK, Jacob S, Sullivan R, Barton M. Evidence-based benchmarks for use of cancer surgery in high-income countries: a population-based analysis. *Lancet Oncol*. Lancet Publishing Group; 2021 Feb 1; **22**: 173–181.

56 Finks JF, Osborne NH, Birkmeyer JD. Trends in Hospital Volume and Operative Mortality for High-Risk Surgery. *New England Journal of Medicine*. Massachusetts Medical Society; 2011 Jun 2; **364**: 2128–2137.

57 Gooiker GA, Van Der Geest LGM, Wouters MWJM, Vonk M, Karsten TM, Tollenaar RAEM, *et al.* Quality improvement of pancreatic surgery by centralization in the Western Part of the Netherlands. *Ann Surg Oncol*. 2011 Jul; **18**: 1821–1829.

58 Sánchez-Velázquez P, Muller X, Malleo G, Park JS, Hwang HK, Napoli N, *et al.* Benchmarks in Pancreatic Surgery: A Novel Tool for Unbiased Outcome Comparisons. *Ann Surg*. Lippincott Williams and Wilkins; 2019 Aug 1; **270**: 211–218.

59 Uesaka K, Boku N, Fukutomi A, Okamura Y, Konishi M, Matsumoto I, *et al.* Adjuvant chemotherapy of S-1 versus gemcitabine for resected pancreatic cancer: a phase 3, open-label, randomised, non-inferiority trial (JASPAC 01). *The Lancet* [Internet]. Elsevier; 2016 Jul 16; **388**: 248–257. Available from: https://doi.org/10.1016/S0140-6736(16)30583-9

60 Conroy T, Ychou M, Bouché O, Guimbaud R, Bécouarn Y, Adenis A, *et al.* FOLFIRINOX versus Gemcitabine for Metastatic Pancreatic Cancer. *New England Journal of Medicine*. 2011; **364**: 1817–1842.
