# Supplementary figures and images for: Relationship between pancreatic cancer resection rate and survival at population level: systematic review
Source: BJS Open. 2025 Mar 25;9(2):zraf007. doi: 10.1093/bjsopen/zraf007 (PMC11934921; doi:10.1093/bjsopen/zraf007)

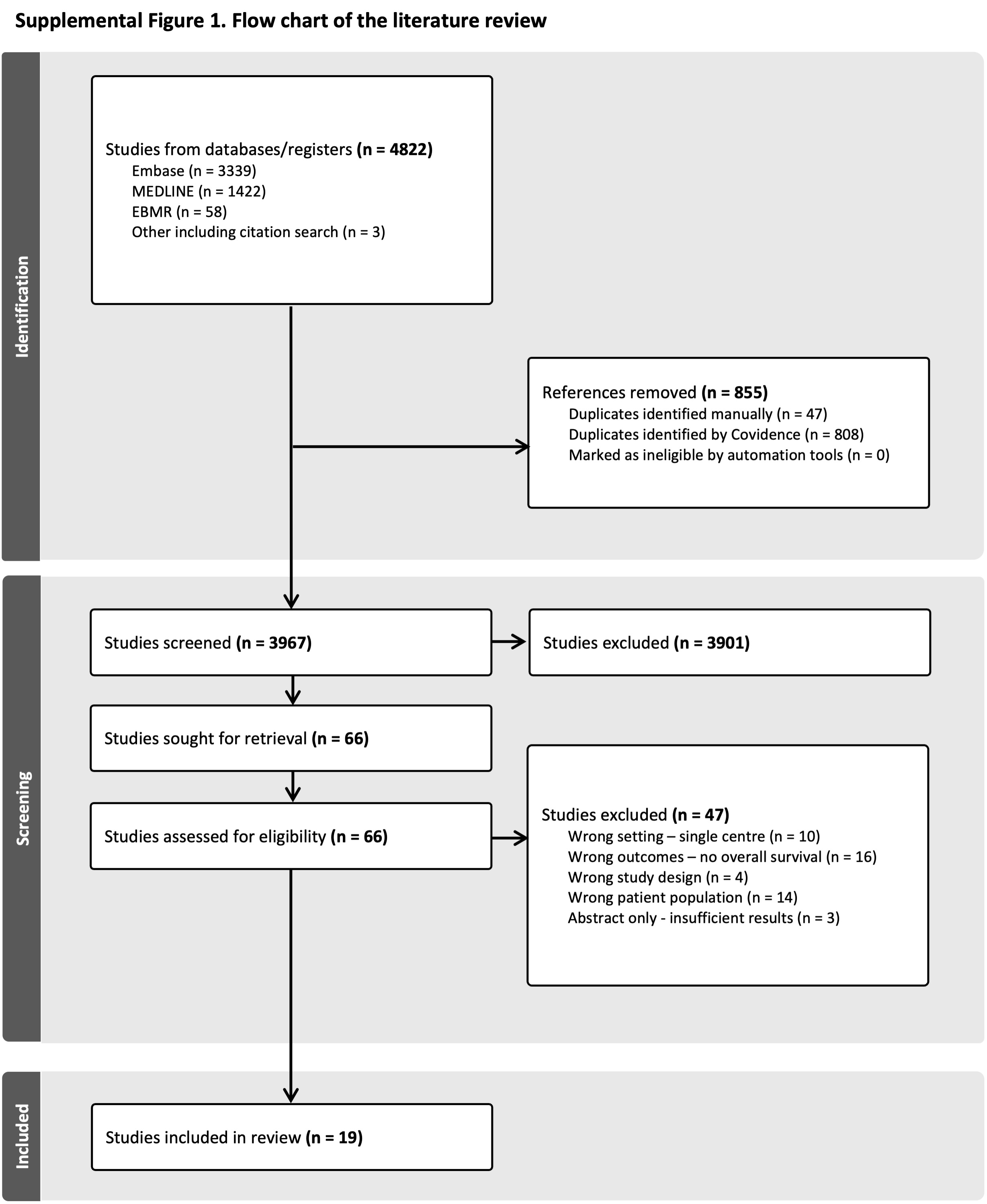

Supplement: zraf007_Supplementary_Data [file zraf007_supplementary_data.zip › PRISMA flow chart.jpg]
